# Peripheral Cone Dystrophy: Expanded Clinical Spectrum, Multimodal and Ultrawide-Field Imaging, and Genomic Analysis

**DOI:** 10.1155/2018/2984934

**Published:** 2018-07-11

**Authors:** Robert A. Sisk, Robert B. Hufnagel, Ailee Laham, Elizabeth S. Wohler, Nara Sobreira, Zubair M. Ahmed

**Affiliations:** ^1^Department of Ophthalmology, University of Cincinnati College of Medicine, Cincinnati, OH, USA; ^2^Cincinnati Eye Institute, Cincinnati, OH, USA; ^3^Division of Pediatric Ophthalmology, Cincinnati Children's Hospital Medical Center, Cincinnati, OH, USA; ^4^Division of Human Genetics, Cincinnati Children's Hospital Medical Center, Cincinnati, OH, USA; ^5^McKusick-Nathans Institute of Genetic Medicine, Johns Hopkins University School of Medicine, Baltimore, MD, USA; ^6^Department of Otorhinolaryngology, School of Medicine, University of Maryland, Baltimore, MD, USA

## Abstract

**Purpose:**

To present new clinical features, multimodal and ultrawide-field imaging characteristics of peripheral cone dystrophy (PCD), and results of laboratory and genetic investigation to decipher the etiology.

**Methods:**

Retrospective observational case-series.

**Results:**

Three patients with PCD presented with bilateral paracentral scotomas and a mean visual acuity of 20/25. All exhibited confluent macular hyperautofluorescence with a central bull's eye lesion. Spectral-domain optical coherence tomography revealed loss of outer retinal elements, particularly the inner segment ellipsoid band and external limiting membrane, within the area of macular hyperautofluorescence. This area corresponded with a lightened fundus appearance and variable retinal pigment epithelium (RPE) abnormalities. Full field and multifocal electroretinography distinguished PCD from other photoreceptor dystrophies. Ultrawide-field imaging revealed irregular peripheral retinal lesions in a distribution greater nasally than temporally and not contiguous with the macular lesion. Functional and anatomic testing remained stable over a mean follow-up of 3 years. Laboratory investigation for causes of uveitis was negative. Whole exome sequencing identified rare variants in genes associated with macular or cone dystrophy or degeneration.

**Conclusions:**

In contrast to the original description, the funduscopic and fluorescein angiographic appearance of PCD is abnormal, although the defects are subtle. Peripheral lesions may be observed in some patients. Bilateral, symmetric, macular hyperautofluorescence associated with outer retinal atrophy that spares the fovea is a characteristic of PCD. Pathogenic variants in the same gene were not shared across the cohort, suggesting genetic heterogeneity. Further evaluation is warranted.

## 1. Introduction

Cone dystrophy is a slowly progressive, diffuse photoreceptor dystrophy that presents as hemeralopia, reduced visual acuity, and nystagmus associated with macular cone photoreceptor and retinal pigment epithelium (RPE) atrophy [1–6]. Two forms of localized cone dysfunction syndromes have been described: occult macular dystrophy (OCMD; MIM 613587) and peripheral cone dystrophy (PCD; MIM 609021) [7–16]. OCMD and PCD can be segregated by electrophysiologic responses to full field electroretinography (ffERG) and multifocal electroretinography (mfERG). OCMD displays normal photopic waveforms on ffERG and reduced mfERG responses only at the fovea, which correlates clinically with reduced visual acuities and foveal cone photoreceptor atrophy seen on spectral-domain optical coherence tomography (SDOCT). OCMD is inherited in a dominant fashion and is usually associated with mutations in *RP1L1* [17].

PCD is a very rare retinal disease originally characterized by normal fundoscopic appearance, normal fluorescein angiographic imaging, mildly reduced photopic potentials with preserved scotopic potentials on ffERG, and relative preservation of foveal cone function by mfERG. The name “peripheral cone dystrophy” is unfortunately a misnomer, as central rather than peripheral cone involvement is a prominent feature, but the name was selected to contrast “central” cone dystrophy, or OCMD, because the foveola is preserved. Kondo and Miyake, who also first described OCMD, first reported three cases of PCD, who presented with bilateral ring scotomas and near-normal visual acuity [7, 12]. These seminal cases included a pair of affected siblings, suggesting autosomal recessive inheritance or autosomal dominant inheritance with parental germline mosaicism. However, no genetic cause has been associated with this disorder.

PCD is a diagnosis of exclusion. The differential diagnosis includes Stargardt disease, cone dystrophy, enhanced S-cone syndrome, pericentral retinitis pigmentosa, syphilitic placoid chorioretinitis, acute zonal occult outer retinopathy (AZOOR), posterior scleritis, traumatic retinopathy (commotio retinae), posterior uveitis, hydroxychloroquine toxicity, and autoimmune retinopathy [18–22]. Here, we report previously undescribed funduscopic, fundus autofluorescence (FAF), infrared imaging, fluorescein angiography (FA), and SDOCT abnormalities in three probands with PCD. We also describe results of our investigation using whole exome sequencing (WES) to identify a causative gene among our three unrelated probands.

## 2. Methods

### 2.1. Human Subjects Research

Institutional Review Board (IRB) approval was obtained for retrospective and prospective evaluation of all cases of PCD diagnosed at the Cincinnati Eye Institute between September 1, 2009, and May 1, 2013. Records were reviewed for patient age, sex, demographics, family history, medical history, toxic and infectious exposures, and ocular examination findings. Patients underwent extensive ophthalmic testing including fundus imaging with standard (TRC-50DX®, Topcon Medical Systems, Oakland, NJ) and ultrawide-field cameras (Optos 200Tx®, Optos, Marlborough, MA). Multimodal imaging (Spectralis® HRA-OCT, Heidelberg Engineering, Dossenheim, Germany) was utilized including red-free imaging, infrared imaging (IR), FAF, FA, and SDOCT. Full field and multifocal electroretinography (Diagnosys D218, Software V6.0.47 with 61 hexagon array, Lowell, MA) was performed to meet ISCEV standards. B-scan ultrasonography (Eye Cubed I^3^® unit, Ellex, Minneapolis, MN) was performed to exclude posterior scleritis or infiltrative choroidal disease. Laboratory testing was performed for infectious and noninfectious causes of uveitis, including syphilis, tuberculosis, cat-scratch disease, Lyme disease, toxoplasmosis, ANCA-associated uveitides, systemic lupus erythematosus, and sarcoidosis. HLA testing was performed to evaluate for HLA-B27-associated uveitis and birdshot chorioretinopathy.

### 2.2. Molecular Genetic Investigation

After informed consent was obtained to participate in genetic research, whole blood samples were obtained from all patients. Genetic analysis was performed through the BHCMG and the National Eye Institute (eyeGENE®, National Ophthalmic Genotyping and Phenotyping Network, Stage 1—Creation of DNA Repository for Inherited Ophthalmic Diseases). Sanger sequencing for *ABCA4*, *PRPH2*, and *ELOVL4* was performed on one proband (case 1), and *PRPH2*/RDS-peripherin was sequenced in all cases. The Baylor-Hopkins Center for Mendelian Genomics (BHCMG) performed WES and analysis on all three probands. The aforementioned testing, clinical histories, and family histories were provided to these organizations with the patients' permission using PhenoDB [23].

We used WES to investigate both for variations in retinal dystrophy genes in each proband and for genes commonly mutated in the 3 affected individuals. The Agilent SureSelect HumanAllExonV4_51MbKit_S03723314 was used for exome capture. Libraries were sequenced on the HiSeq2500 platform with onboard clustering using 100 bp paired end runs and sequencing chemistry kits TruSeq Rapid PE Cluster Kit-HS and TruSeq Rapid SBS-HS. FASTQ files were aligned with BWA [24] version 0.5.10-tpx to the 1000 genomes phase 2 (GRCh37) human genome reference. Duplicate reads were flagged with Picard version 1.74. Local realignment around indels and base call quality score recalibration were performed using the Genome Analysis Toolkit (GATK) 2.3–9 multisample calling with a Unified Genotyper [25]. Variant filtering was done using the variant quality score recalibration (VQSR) method [26]. The variant prioritization strategy was designed using the Variant Analysis Tool of PhenoDB [27] and Ingenuity Variant Analysis (Qiagen, Redwood City, CA). Rare functional variants (missense, nonsense, splice site variants, and indels) with a MAF ≤0.01 in the Exome Variant Server (release ESP6500SI-V2) or 1000 Genomes Project were prioritized [28]. We also excluded all variants found in in-house controls for PhenoDB (CIDRVar 51 Mb) and Cincinnati Children's Hospital Medical Center (CCHMC). Postanalysis, PCR primers were designed to amplify exons and flanking intronic splice sites followed by direct Sanger sequencing to validate the candidate causative variants.

## 3. Results

### 3.1. Case Descriptions

Three patients (two female and one male) were identified at a median age of 51 years (range 42 to 57 years) with visual symptoms that began at a mean of 9 years prior. Mean best-corrected visual acuity was 20/25, and it remained stable throughout the mean follow-up of 3 years. All complained of paracentral visual field loss and mildly reduced best-corrected visual acuities, and no patient described progression beyond their initial presentation. No patient could attribute a precipitating event, injury, or illness to the onset of symptoms, yet none described their vision loss as acute. Only case 2 had any family history of retinal dystrophy or blindness or exposure to any medication known to be toxic to the retina or RPE. Laboratory investigation for causes of infectious and noninfectious uveitis was negative in all patients.

#### 3.1.1. Case 1

**Figure 1:**
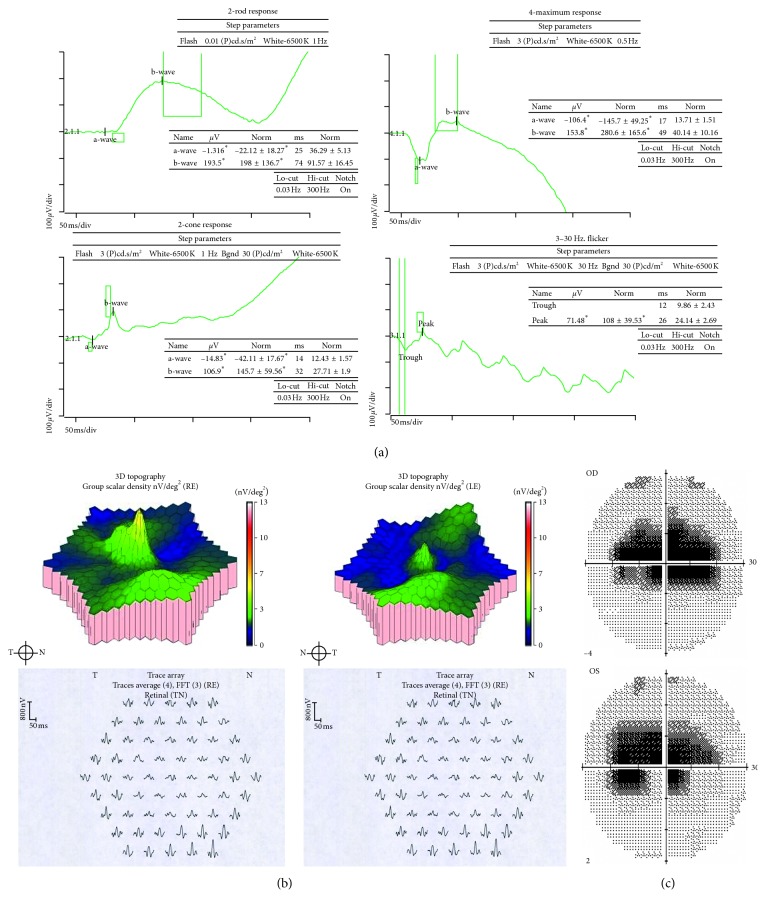
Full field electroretinography (ffERG), multifocal electroretinography (mfERG), and visual field testing of case 1. (a) Full field electroretinography demonstrated low normal a- and b-wave amplitudes and implicit times on scotopic testing. Photopic testing showed moderately reduced a- and b-wave amplitudes with mildly delayed implicit times. See Supplementary Figure 1 for normal reference and ffERG for cases 2 and 3. (b) Central amplitudes were reduced in the central and paracentral 15° with relative sparing of the fovea on multifocal electroretinography. See Supplementary Figure 2 for normal reference and mfERG testing for cases 2 and 3. (c) Humphrey Sita-Standard protocol with size III stimulus revealed bilateral dense paracentral scotomas that spared foveal sensitivities. See Supplementary Figure 3 for visual field testing of cases 2 and 3.

A 57-year-old Caucasian male presented with a ten-year history of nonprogressive ring scotomas OU (Figure 1). He denied other ocular- or nonocular-associated symptoms or any prior ocular trauma. Family history was negative for any retinal disease, uncorrectable vision loss, hemeralopia, or nystagmus. Best-corrected visual acuities on presentation were 20/25-2 OU. Ishihara color vision testing was diminished to four out of eleven plates in each eye. Anterior segment examination was unremarkable except for mild nuclear sclerotic cataracts in both eyes. He exhibited typical funduscopic findings for pathologic myopia including staphylomatous changes, parapapillary atrophy, and inferotemporal lacquer cracks in the left eye and areas of chorioretinal atrophy in both the posterior pole and periphery OU.

#### 3.1.2. Case 2

A 52-year-old African American female was referred for evaluation after three years of hydroxychloroquine treatment for rheumatoid arthritis. The medication dosage was never supratherapeutic, and she denied visual changes on the medication. Interestingly, her visual complaints predated the use of the medication by two years, but no baseline visual field testing had been performed. She described her mother as having “macular degeneration and retinitis pigmentosa” that began as central vision loss in her forties and progressed to nyctalopia and peripheral vision loss. Visual acuities were 20/20 OU, and anterior segment examination was unremarkable. Ishihara color vision testing was diminished to ten out of fifteen plates in the right eye and eleven out of fifteen plates in the left eye. The right eye had received laser retinopexy after posterior vitreous detachment for symptomatic retinal holes associated with lattice degeneration.

#### 3.1.3. Case 3

A 42-year-old Caucasian female who originally presented 22 years prior with perimacular pigmentary changes had been diagnosed with bilateral choroidal osteomas, although neither eye had an orange choroidal lesion nor hyperreflective plaque by B-scan ultrasonography on any prior testing. She denied progression of vision loss, although visual acuities at original presentation were 20/20 OD and 20/30 OS and declined to 20/30 OU when diagnosed with PCD. Ishihara color vision testing was diminished to three out of fifteen plates in each eye. Her family history was negative for eye-related phenotypes. Anterior segment examination was normal, but fundus examination showed perimacular arcuate and circumferential nasal retinal lightening with central pigmentary clumping OU.

### 3.2. Electroretinography

Full field electroretinography (ffERG) showed a pattern consistent with cone dystrophy for cases 1 and 3 (Supplementary Figure 1). There were relatively preserved (at the lower limit of our normal reference range) a- and b-wave amplitudes with normal implicit times on scotopic testing and moderately reduced amplitudes with mildly increased implicit times on photopic testing. ffERG resembled cone-rod dystrophy for case 2 with moderate reductions in a- and b-wave amplitudes and delayed implicit times on scotopic and photopic testing (Supplementary Figure 2). Responses were symmetrical between OD and OS in most recorded waveforms in all patients. Waveforms had otherwise typical architecture and specifically did not have a sinusoidal appearance. Multifocal electroretinography demonstrated diffusely reduced amplitudes and increased implicit times with relative sparing of the foveal spike OU (Supplementary Figure 2).

### 3.3. Visual Fields

Humphrey 30-2 Sita Standard and Goldmann threshold visual field testing confirmed bilateral paracentral scotomas in all patients that remained stable throughout follow-up (Supplementary Figure 3). The macular appearance was unremarkable funduscopically in all cases except for the myopic fundus changes in case 1. Peripheral RPE alterations were observed in the nasal peripheral retina in all patients. These were unilateral in case 2 and attributable to a history of laser retinopexy for retinal breaks associated with posterior vitreous detachment. Scotomas were not observed corresponding to these peripheral lesions.

### 3.4. Blue-Light Fundus Autofluorescence

**Figure 2:**
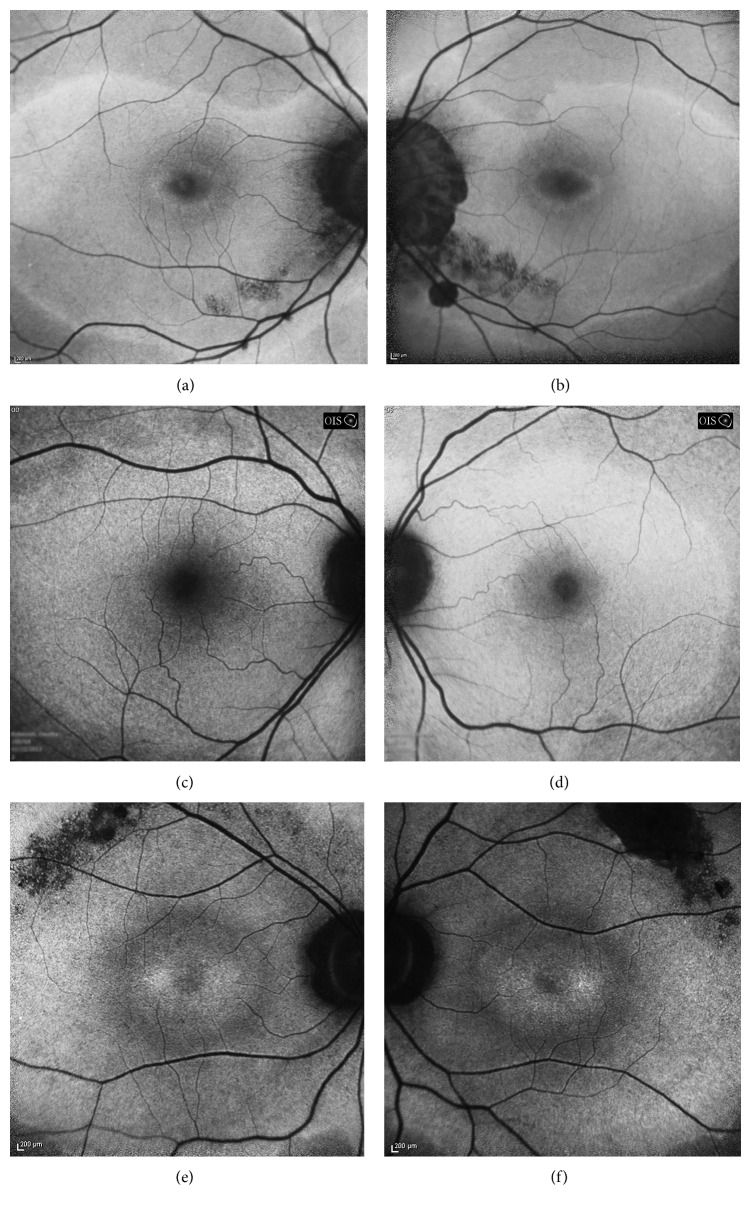
Thirty degree confocal scanning laser ophthalmoscope-based fundus autofluorescence by 488 nm argon blue laser excitation and emission filtered less than 500 nm. (a, b) Right and left maculas of case 1 had a geographic area of hyperautofluorescence with uniform hyperautofluorescent scalloped border that encompassed the majority of the macula and parapapillary retina outside alpha zone atrophy (seen as intensely hypoautofluorescent). A central ovoid bull's eye lesion of alternating hyper- and hypoautofluorescence was centered on the fovea. Linear granular hypoautofluorescence from lacquer cracks was observed in this patient with high myopia. (c, d) Right and left maculas of case 2 showed similar features, except the outer hyperautofluorescent border and bull's eye lesions were not as prominent. (e, f) Right and left maculas of case 3 also exhibited coarse, densely hypoautofluorescent arcs along the superotemporal arcades and a larger central bull's eye lesion in each eye.

**Figure 3:**
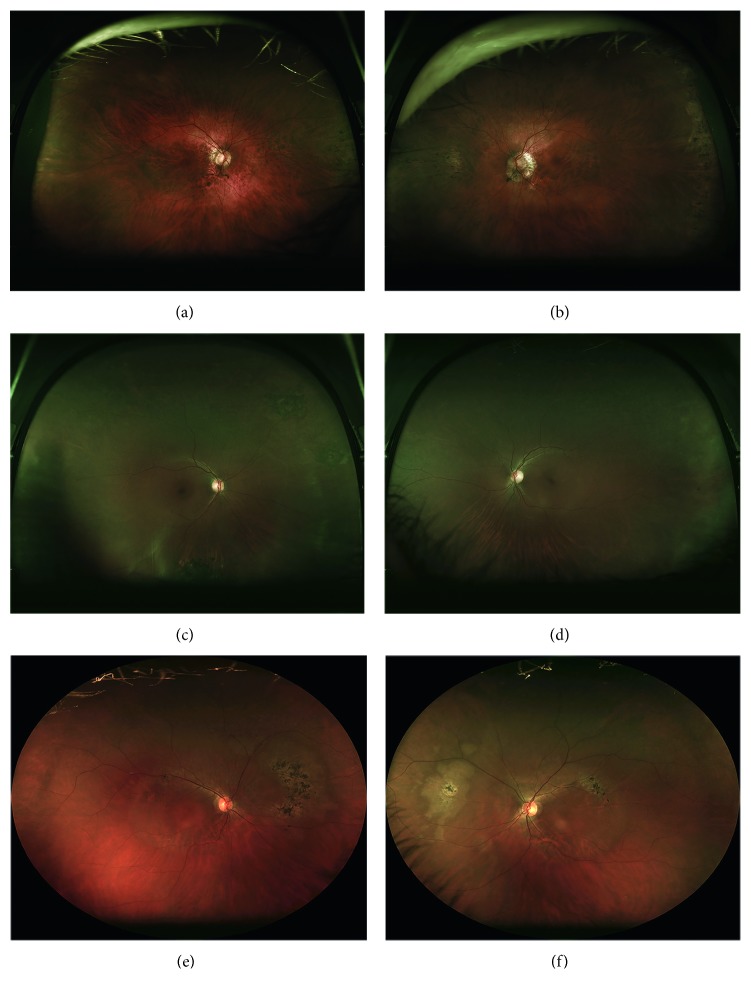
Two hundred degree ultrawide-field color retinal imaging using 633 nm, 532 nm, and 488 nm lasers demonstrate subtle retinal whitening in all patients corresponding to the geographic areas of hyperautofluorescence observed in Figure 2. Peripheral retinal pigment epithelial abnormalities were seen in the nasal periphery in all patients, although these were attributable to prior laser retinopexy in case 2. (a, b) Right and left fundi of case 1 exhibited alpha zone parapapillary atrophy and surrounding fundus lightening associated with high myopia. A nasal tongue of pigmentary changes extended from the peripheral retina posteriorly along the horizontal midline in each eye. (c, d) Right and left fundi of case 2 showed areas of lattice degeneration in both eyes surrounded by chorioretinal scarring from laser treatment. (e, f) Right and left fundi of case 3 had prominent posterior rings of retinal whitening and pigmentary alterations centered around the maculas. Irregular circumferential lesions with central reticular intraretinal pigment migration were observed nasally in both eyes.

All probands had bilateral, relatively symmetric, central, geographic areas of confluent macular hyperautofluorescence with rounded or scalloped borders that extended nasally past the optic disc (Figure 2). In cases 1 and 3, this area was sharply delineated from the surrounding isoautofluorescence by a narrow border of hyperautofluorescence of greater intensity than the central confluence. This transition could be observed funduscopically by a subtle color change best appreciated on the laser-generated ultrawide-field images (Figure 3). Case 2 had a gradual transition in FAF and no funduscopically visible transition. The caliber of retinal vessels was reduced with the areas of central FAF alterations in all patients. Focal curvilinear hypoautofluorescent interruptions were present in two probands with funduscopically visible RPE alterations (lacquer cracks of high myopia in case 1 and a superior, symmetrical, serpiginous band of RPE thinning and clumping extending away from the disc in case 3). Centrally, a bull's eye lesion of alternating rings centripetally of hypoautofluorescence and hyperautofluorescence surrounding normal foveal hypoautofluorescence was observed in all patients, although the bull's eye lesion was least distinct in case 2.

**Figure 4:**
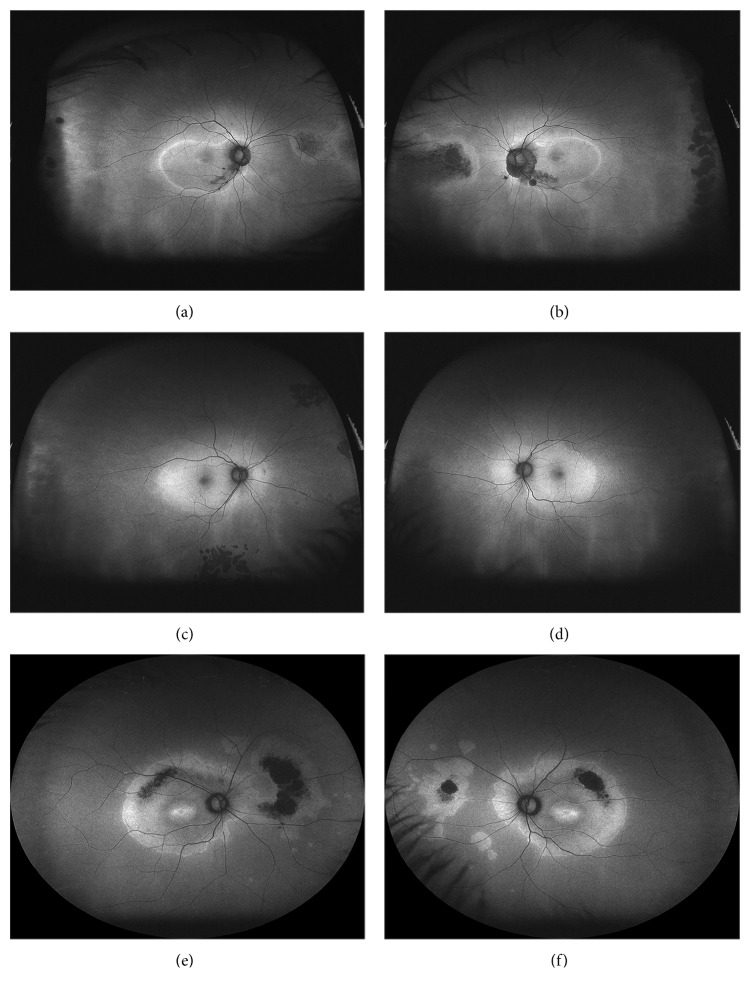
Two hundred degree ultrawide-field fundus autofluorescence with 488 nm laser excitation demonstrates macular changes as described in Figure 2 but reveals additional peripheral autofluorescence abnormalities in all patients. (a, b) Right and left fundi of case 1 had grouped nummular hypoautofluorescent areas anterior to the equator from cobblestone degeneration. A hypoautofluorescent tongue with hyperautofluorescent borders extended posteriorly towards the optic nerve in both eyes. Areas of funduscopically visible black pigment clumping appeared densely hypoautofluorescent. (c, d) Hypoautofluorescence from prior laser treatment of lattice degeneration in the right eye was the only evident peripheral autofluorescence abnormality seen in case 2. (e, f) A bifocal area of hyperautofluorescence with central hypoautofluorescence to the nasal portion was observed in the right and left fundi of case 3. In the right eye, the lesions were contiguous and the area of hypoautofluorescence was larger.

Peripheral autofluorescence abnormalities were appreciated on ultrawide-field imaging in cases 1 and 3 (Figure 4). These ranged from clustered patches of arcuate, amoeboid hyperautofluorescence with variable central hypoautofluorescence to discrete, isolated ovoid areas of hyperautofluorescence of variable size. The location of peripheral lesions was variable but tended to involve the nasal hemiretina, producing a bifocal appearance when viewed against the macula on the ultrawide-field images. Funduscopically, this corresponded to lightened areas of the nasal fundus (cases 1 and 3) with central RPE pigment clumping and intraretinal migration (case 3).

### 3.5. Fluorescein Angiography

FA demonstrated a subtle hyperfluorescence at the area of confluent macular hyperautofluorescence. Areas of funduscopically visible RPE thinning and atrophy demonstrated anticipated window defects. Areas of RPE clumping exhibited blockage. No patient had vascular filling defects or leakage. Case 2 had blocked choroidal fluorescence. Interestingly, case 1, who had a single pathologic *ABCA4* mutation, did not have blocked choroidal fluorescence.

### 3.6. Infrared Imaging

Infrared imaging was less useful for discerning the transition from abnormal to intact outer retinal architecture, although there was a subtle intensity change that mirrored the transition zone on FAF imaging.

### 3.7. Ultrasonography

B-scan ultrasonography excluded posterior scleritis, choroidal thickening or infiltration, or retrobulbar disease (data not shown).

### 3.8. Spectral-Domain Optical Coherence Tomography

**Figure 5:**
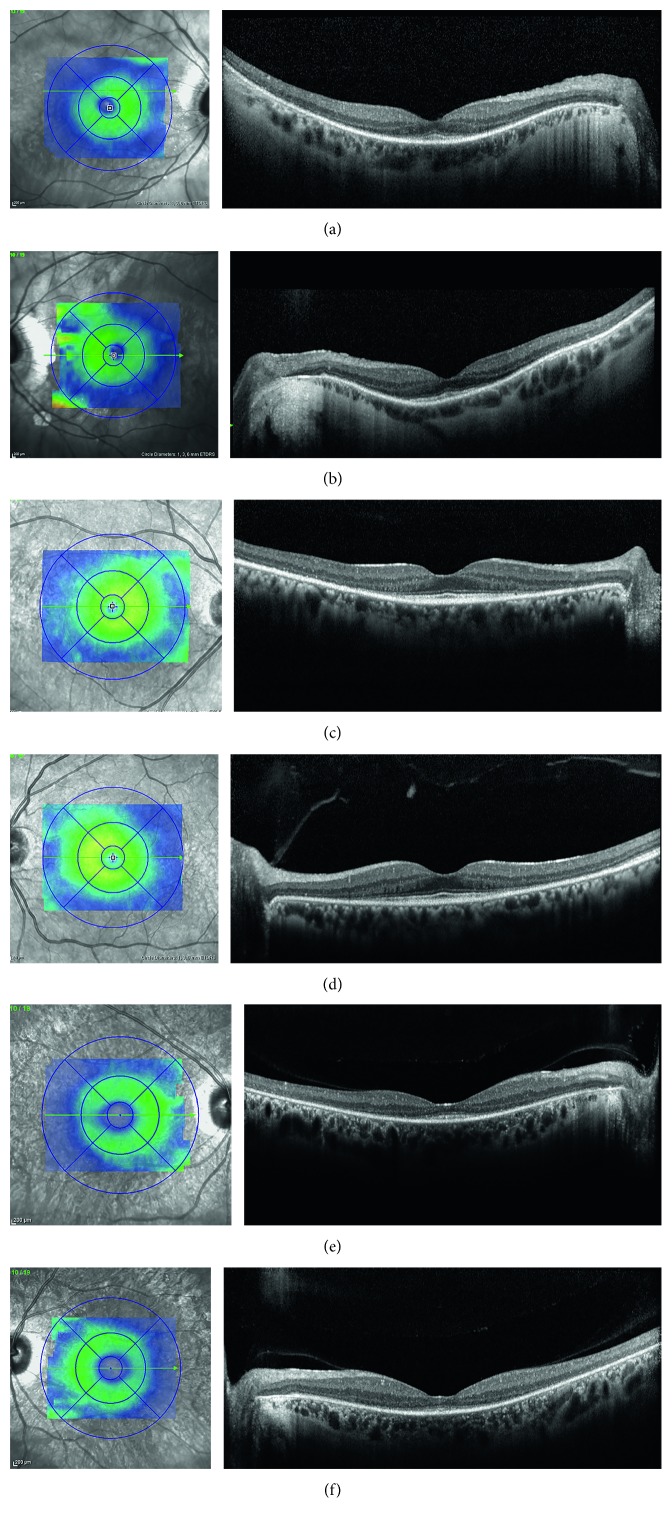
Spectral-domain optical coherence tomography registration thickness maps (left column) exhibited severe retinal thinning in all eyes. Accompanying foveal horizontal raster scans (right column) demonstrated outer retinal loss sparing the foveola in both eyes of all patients. The external limiting membrane, inner segment ellipsoid band, and photoreceptor outer segments were lost centrifugally until the hyperautofluorescent border of the macular lesions in Figure 2, where there was transition to normal retinal architecture. All cases had normal choroidal thickness, except case 1, who had pathologic myopia. (a, b) Right and left maculas of case 1 had staphylomatous posterior pole curvature, alpha zone parapapillary RPE atrophy, and choroidal thinning associated with pathologic myopia. (c, d) Right and left maculas of case 2 had the greatest preservation of outer retinal layers at the fovea and the nerve fiber layer throughout the macula compared to the other two cases. (e, f) Right and left maculas of case 3 had reduced inner retinal thickness with preservation of inner retinal lamination, similar to case 1.

SDOCT demonstrated reduced macular thickness and volume measurements in all probands due to loss of outer retinal elements (Figure 5). Inner retinal thickness and architecture were undisturbed. The inner segment ellipsoid (iSE) band, external limiting membrane (ELM), and even photoreceptor cell bodies were diminished or absent within the well-defined area of confluent macular hyperautofluorescence. The external border between hyperautofluorescence and isoautofluorescence marked the transition from abnormal to intact outer retinal architecture and lamination. The central bull's eye lesion on FAF imaging represented a transition from diminished to intact iSE and ELM bands, a pattern previously observed with other photoreceptor dystrophies. Within the macula, retinal architecture was most intact at the fovea, consistent with the sparing of central visual acuities relative to the surrounding paracentral scotomas. The choroid was normal in thickness, except for an anticipated amount of thinning in case 1 related to high myopia.

### 3.9. Genetic Analysis

Genetic testing for genes associated with cone dysfunction, retinitis pigmentosa, or lipofuscin accumulation revealed no pathogenic variants in a pattern consistent with a monogenic cause for disease. Specifically, Sanger sequencing of *ABCA4*, *PRPH2*/RDS-peripherin, and *ELOVL4* was negative except for Proband 1, who had a heterozygous *ABCA4* missense variant (c.2588G>C; p.Gly863Ala) and no family history of Stargardt disease or cone-rod dystrophy. This substitution has been previously reported as disease-causing (rs76157638; HGMD ID CS024003), but not associated with pericentral retinal degeneration (PRD) [29–32].

WES was then performed on the three probands. Details of bioinformatics analysis are available in Methods. Briefly, we prioritized rare functional variants (missense, nonsense, splice site variants, and indels) that were heterozygous, homozygous, or compound heterozygous in each of the 3 probands and excluded variants with a MAF >0.01 in the Exome Variant Server (release ESP6500SI-V2), 1000 Genomes Project, or Exome Aggregation Consortium (ExAC) [28, 33]. We also excluded all variants with a frequency of >0.01 found in in-house controls for BHCMG (CIDRVar 51 Mb) and CCHMC. This allele frequency cutoff was used to account for variants causing autosomal recessive disease. Parent and sibling samples were not available for segregation analysis.

Variant lists generated for each proband were first screened for rare variants (<1% minor allele frequency) in 52 genes related to pattern dystrophy, macular dystrophy, cone dystrophy, cone-rod dystrophy, rod-cone dystrophy, and cone photoreceptor development and function, including cone opsin genes (Supplementary Table 1). Supplementary Table 2 lists the 7 variants that were validated by Sanger sequencing. The *ABCA4* variant in case 1 was also detected by the WES. In cases 1 and 2, missense variants were identified in *IMPG2*, p.Leu842Met, and p.Ser11Tyr, respectively. *IMPG2* is associated with autosomal dominant vitelliform macular dystrophy 5 [MIM #616152] [34]. Both variants were predicted pathogenic by SIFT and Polyphen-2 [35, 36]. In case 2, a rare, predicted-pathogenic variant was noted in the protein kinase domain of *GUCY2D* (p.Glu779Lys), associated with autosomal dominant cone-rod dystrophy 6 [MIM #601777] [37, 38]. Additionally in case 2, we identified two variants in *RP1L1*, p.Ala624Thr, and p.Trp2306Arg. However, neither were predicted pathogenic by Polyphen-2, and only p.Trp2306Arg was predicted pathogenic by SIFT. In case 3, we identified one predicted-pathogenic variant in *ADAM9* (p.Gln800His), associated with cone-rod dystrophy 9 [MIM #612775] [39]. We then searched for genes with rare variants in other retinal dystrophy genes (Supplementary Table 3) and rare variants in all 3 probands (not shown), though no common candidate gene was identified with rare variants. These variants are all considered variants of uncertain significance (VOUSs).

## 4. Discussion

At the time of its initial description a decade ago, PCD was an electrophysiologic diagnosis in individuals with normal fundus appearance and fluorescein angiography [12]. OCT and FAF had limited clinical applications and lacked the resolution available with conventional scanning laser ophthalmoscope-based platforms. Yet review of the fundus photography and fluorescein angiography images from the report by Kondo and coauthors demonstrates the same features we describe in our cohort: (a) a lightened macular color funduscopically that transitions at the temporal arcades, (b) narrowing of retinal vessels within the affected macular region, (c) macular hyperfluorescence on FA that transitions at the same location as the funduscopic color change, and (d) variable blocked choroidal fluorescence outside the macular lesion. In our cohort, two of the three probands also had nasal RPE and retinal changes that were not described in the original report. From the findings presented, we assert that PCD has features on ophthalmoscopy and multimodal imaging that distinguishes this diagnosis from other rare diseases.

SDOCT and FAF clarify the functional deficits observed by visual field and electroretinographic testing. The total macular volume is reduced, and the photoreceptor layer within the macular lesion, except of the fovea, is thinned and disorganized. The iSE and ELM bands particularly are diminished or absent with greater involvement of cone photoreceptor cell bodies centrifugally until the border of the hyperautofluorescent lesion. This unveiling of outer retinal elements was associated with the observed window defect of confluent macular hyperautofluorescence [40]. The transition between affected and unaffected outer retina correlated well with the transition from hyperautofluorescence to isoautofluorescence. Unlike SDOCT changes observed in retinitis pigmentosa and typical cone-rod dystrophies, choroidal thickness was not reduced, and the RPE remained largely intact except focal disruptions which were also evident funduscopically [41–49]. These findings corroborate that the photoreceptor layer is primarily affected, and the RPE may be secondarily affected. Ultrawide-field FAF imaging revealed large affected regions nasally that produced a bifocal appearance and helped explain the greater reduction in cone amplitudes than would be expected for a purely macular disease. Despite no reduction in inner retinal thickness by SDOCT, retinal arterioles within the central hyperautofluorescent region with photoreceptor disease were thinned. There was no evidence from SDOCT or FA imaging of a primary vascular disease. Without photoreceptor-generated impulses from affected areas, presumably, there is a reduced activity of inner retinal layers, reduced oxygen demand, and a resultant reduction in vessel caliber by autoregulation. This has been observed commonly in other photoreceptor dystrophies [50]. We observed greater variation in full field electroretinographic findings than the original series presented by Kondo et al., with cases 1 and 3 displaying the typical pattern reported for PCD and case 2 having a cone-rod dystrophy pattern.

PCD can be distinguished from pericentral retinal degeneration (PRD), which shares many clinical features, and has heterogeneous genetic causes, particularly genes causing retinitis pigmentosa and Stargardt disease. Foremost, the electrophysiologic profile for PRD resembles rod-cone dystrophy rather than cone or cone-rod dystrophy. Annular visual field loss is complete in PRD and may be incomplete in PCD. Peripheral retinal pigmentary changes are not observed in PRD, so annular or curvilinear visual field deficits are not observed beyond forty degrees, as are seen in our cases of PCD. Cases of *ABCA4*-related PRD involve mutations that prevent production of a protein product and produce photoreceptor dysfunction. Heterozygous mutations in ABCA4 can produce bull's eye lesions or occult macular dystrophy, but photoreceptor loss does not extend past the arcades and is accompanied by progressive RPE atrophy.

PCD may be a nonspecific rare presentation of cone dystrophy, and this is supported by pathogenic variants associated with macular dystrophy in all patients without a consensus candidate gene among them. Although WES did not reveal the leading candidate gene(s) among the probands, all probands harbored VOUSs in known photoreceptor dystrophy genes. We observed rare and predicted pathogenic variants in *IMPG2* in cases 1 and 2, which is associated with autosomal dominant vitelliform macular dystrophy, and variants in *RP1L1* in case 2, which is associated with autosomal dominant occult macular dystrophy [17, 34]. Both genes cause a variable macular phenotype overlapping with peripheral cone dystrophy. The manifestations of *RP1L1* pathogenic variants are complex and include both limited and diffuse forms of cone dystrophy [17, 51, 52]. We speculate that *RP1L1* may modulate the effects of another cone dystrophy gene or autoimmune retinopathy contributing to allow sparing of the foveolar cones in the PCD phenotype. In case 2, the previously unreported variant in *GUCY2D*, associated with autosomal dominant cone-rod dystrophy, was considered as potentially contributing to this phenotype given the cone-rod pattern on ffERG and her mother's end stage cone-rod dystrophy phenotype. Given foveal sparing in both case 2 and her mother, the phenotype may be modulated by the *RP1L1* variant. No prior report of *GUCY2D* or *ADAM9*-related cone dystrophy involves broad macular involvement sparing the foveola [39, 53–55]. Rather, those diseases typically involve the fovea early and have prominent macular RPE changes, and those with bull's eye lesions sparing the umbo do not extend beyond the parafovea.

Several features of PCD could also be consistent with postinflammatory changes of a retinal white dot syndrome [19, 56–65]. The geographic and racial backgrounds were variable, and two cases were sporadic. The regional clustering of both cohorts in the peer-reviewed literature against time and geography favors an autoimmune disease incited by a local pathogen or toxin. The lack of progression observed by anatomical and electrophysiological testing is atypical for retinal dystrophy, although follow-up with high-resolution anatomic testing was performed after three years and may represent the end stage of disease progression. Case 3 was noted to have nonprogressive symptoms and stable fundus appearance when compared with her original retinal drawings from twenty years prior. The Japanese cohort has exhibited similar stability after the publication (Kondo, *personal communication* 2013). The variable involvement of the peripheral retina resembles the random pattern observed in inflammation more than the predictable patterns observed in retinal dystrophies. The peripheral hyperautofluorescent lesions with central hypofluorescent RPE disruption are similar to those observed in other retinal white dot syndromes (MEWDS, AZOOR, and PIC). On the contrary, specific sparing of the fovea while the surrounding outer retina is decimated would be unexpected for an inflammatory disease. With the exception of MEWDS, severe RPE alterations and persistent scotomas usually accompany preceding chorioretinal inflammation, and these were not observed with the peripheral lesions. The presence of tapetal sheen, seen intermittently in case 2 and not previously described with PCD, is a characteristic for a retinal dystrophy rather than inflammation or drug toxicity.

A third explanation is that both genetic and environmental factors play a role in the development and manifestations of PCD. An environmental agent or pathogen may create an autoimmune response against short wavelength (S-cone) photoreceptors modulated by a pathogenic variant in cone dystrophy genes like *RP1L1*. In case 2, we cannot rule out the contribution of hydroxychloroquine toxicity to her phenotype, although photoreceptor damage to the arcades would be unusual without prominent RPE changes.

In summary, PCD is a distinct clinical entity with ring scotomas, outer retinal attenuation relatively sparing the foveola, macular hyperautofluorescence with bull's eye lesions, and variable peripheral lesions. However, the etiology remains unknown. Studies of human and primate retinas demonstrate absence of S-cone photoreceptors within the foveola, greater S-cone concentrations within the macula than peripheral retina, and greater concentrations in the retinal periphery nasally than temporally [66–68]. This pattern highly correlates with the location of disease observed in our cohort. Given the limitation of patient numbers for this rare disease, further investigation is warranted, including whole exome sequencing of other probands, investigation of trios, animal models, and serum analysis from more recently affected individuals, and a search for an infectious homologue or compound with selective toxicity to S-cone proteins.

## Data Availability

The data used to support the findings of this study are available from the corresponding author upon request.

## Supplementary Materials

Supplementary Materials This article contains additional online-only material. The following should appear online only: Supplementary Tables 1–3. Supplementary Figure 1: results of full field electroretinography of the right eye for the 3 probands and a normal age-matched control. Waveforms were symmetrical between the two eyes of each patient and the control. Supplementary Figure 2: multifocal electroretinography of the right eye for the 3 probands and a normal age-matched control. Waveforms and 3D topography plots were symmetrical between the two eyes of each patient and the control. Supplementary Figure 3: visual field testing for the 3 probands. Humphrey visual field testing using a 30-2 Sita-Standard protocol with a size III stimulus was used for case 1. Cases 2 and 3 were tested using Goldmann threshold perimetry. Paracentral scotomas were identified by the smallest isopter. Case 2 could not discern I2E targets and had constricted I3E within 30°. Case 3 could not discern I3E targets. Supplementary Table 1: candidate photoreceptor and macular dystrophy genes. Supplementary Table 2: variants detected in candidate photoreceptor or macular dystrophy genes. Supplementary Table 3: variants detected in 2 of 3 probands in genes with known retinal expression.
