# Supplementary material for: Peripheral Cone Dystrophy: Expanded Clinical Spectrum, Multimodal and Ultrawide-Field Imaging, and Genomic Analysis
Source: J Ophthalmol. 2018 Jul 11;2018:2984934. doi: 10.1155/2018/2984934 (PMC6079493; doi:10.1155/2018/2984934)
Supplement: Supplementary Materials — This article contains additional online-only material. The following should appear online only: Supplementary Tables 1–3. Supplementary Figure 1: results of full field electroretinography of the right eye for the 3 probands and a normal age-matched control. Waveforms were symmetrical between the two eyes of each patient and the control. Supplementary Figure 2: multifocal electroretinography of the right eye for the 3 probands and a normal age-matched control. Waveforms and 3D topography plots were symmetrical between the two eyes of each patient and the control. Supplementary Figure 3: visual field testing for the 3 probands. Humphrey visual field testing using a 30-2 Sita-Standard protocol with a size III stimulus was used for case 1. Cases 2 and 3 were tested using Goldmann threshold perimetry. Paracentral scotomas were identified by the smallest isopter. Case 2 could not discern I2E targets and had constricted I3E within 30°. Case 3 could not discern I3E targets. Supplementary Table 1: candidate photoreceptor and macular dystrophy genes. Supplementary Table 2: variants detected in candidate photoreceptor or macular dystrophy genes. Supplementary Table 3: variants detected in 2 of 3 probands in genes with known retinal expression. [file 2984934.f1.pdf]

Supplementary Table 1. Candidate photoreceptor and macular dystrophy genes.

**Pattern Dystrophy**	**Cone-Rod Dystrophy**	**Rod-Cone Dystrophy**
ABCA4	*ADAM9*	*CA4*
ATXN7	*AIPL1*	*CERKL*
BEST1	*ALMS1*	*CRB1*
ELOVL4	*BBS10*	*RHO*
IMPG1	*C8orf37*	
IMPG2	*CACNA1F*	
RP1L1	*CDHR1*	
RPGR	*CNNM4*	**Cone Development, Function**
		BLIMP1
	CRX	*NEUROD2*
	DFNB59	*NR2E3*
**Cone Dystrophy**	*GUCY2D*	*NRL*
CACNA2D4		
CNGA3	*PITPNM3*	*OPN1LW*
CNGB3	*POC1B*	*OPN1MW*
GUCA1A	*RAB28*	*OPN1SW*
KCNV2	*RAXL1*	*OTX2*
PDE6C	*RIMS1*	*RAX*
PDE6H	*RPGRIP1*	*RORB2*
PROM1	*SEMA4A*	*RXRG*
PRPH2	*TTLL5*	*THRB2*

Supplementary Table 2. Variants detected in candidate photoreceptor or macular dystrophy genes.

Position	Reference	Variant	Gene	OMIM Disease	Transcript ID	DNA variant	Protein varian	Case 1	Case 2	Case 3	Grantham	PHRED/C-score	SIFT	SIFT Score	PolyPhen-2 Prediction	PhyloP p-value	dbSNP ID	1000 G MAF	EVS MAF	ExAC MAF
Chr1:94517254	C	G	**ABCA4**	**Stargardt disease, Cone-rod dystrophy 3 (recessive)**	NM_000350.2	c.2588G>C	p.G863A	**Het**			60	26.4	Damaging	0.002	Probably Damaging	1.25E-04	rs76157638	0.0012	0.0052	0.0051
Chr3:100962651	G	T	**IMPG2**	**Vitelliform macular dystrophy 5 (dominant)**	NM_016247.3	c.2524C>A	p.L842M	**Het**			15	23.1	Damaging	0	Probably Damaging		rs149896250	0.0006	0.0002	0.0004
Chr3:101039185	G	T	**IMPG2**	**Vitelliform macular dystrophy 5 (dominant)**	NM_016247.3	c.32C>A	p.S11Y		**Het**		144	22.6	Damaging	0	Possibly Damaging		rs137927107	0.0004	0.0003	0.0001
Chr8:10464692	A	T	**RP1L1**	**Occult macular dystrophy (dominant)**	NM_178857.5	c.6916T>A	p.W2306R		**Het**		101	8.732	Damaging	0.02	Benign		rs200403049	0.0026	0.003	0.0009
Chr8:10469738	C	T	**RP1L1**	**Occult macular dystrophy (dominant)**	NM_178857.5	c.1870G>A	p.A624T		**Het**		58	2.405	Tolerated	0.137	Benign		rs141846905	0.0024	0.003	0.0009
Chr8:38961159	G	T	**ADAM9**	**Cone-rod dystrpohy 9 (recessive)**	NM_003816.2	c.2400G>T	p.Q800H			**Het**	24	21.6	Damaging	0.004	Probably Damaging	4.49E-04	rs145276865	0	0.0002	0.00006
Chr17:7917269	G	A	**GUCY2D**	**Cone-rod dystrophy 6 (dominant)**	NM_000180.3	c.2335G>A	p.E779K		**Het**		56	29	Damaging	0.006	Probably Damaging	3.94E-06		0.0004	0	0.000008

Supplementary Table 3. Variants detected in 2 of 3 probands in genes with known retinal expression.

Position	Reference	Sample	Gene	OMIM Function, Disease; Domain	Transcript ID	Transcript Variant	Protein Variant	Case 1	Case 2	Case 3	Grantham	PHRED/C-score	SIFT	SIFT Score	PolyPhen-2	PhyloP p-value	dbSNP ID	1000 G MAF	EVS MAF	ExAC MAF
Chr5:89981639	C	T	**GPR98**	**Usher syndrome 2C (recessive); Calx-beta domain**	NM_032119.3	c.6317C>T	p.A2106V	**Het**			64	27.5	Damaging	0.02	Probably Damaging	5.78E-07	186999408	0.18	0.0036	0.0016
Chr5:90016759	T	C	**GPR98**	**Usher syndrome 2C (recessive); No domain**	NM_032119.3	c.9631T>C	p.S3211P		**Het**		74	22.6	Tolerated	0.09	Probably Damaging			0	0	0.000033
Chr5:90106814	G	A	**GPR98**	**Usher syndrome 2C (recessive); Calx-beta domain**	NM_032119.3	c.15737G>A	p.R5246Q	**Het**			43	24.3	Tolerated	0.44	Possibly Damaging			0	0	0.0000082
Chr6:152720959	C	T	**SYNE1**	**Emery-Dreifuss muscular dystrophy 4 (dominant); Spinocerebellar ataxia 8 (recessive)**	NM_033071.3	c.7030-1G>A	Splice	**Het**			NA	18.06	NA	NA	NA	1.14E-06		0	0	0
Chr6:152847259	C	T	**SYNE1**	**Emery-Dreifuss muscular dystrophy 4 (dominant); Spinocerebellar ataxia 8 (recessive)**	NM_033071.3	c.181G>A	p.V61I		**Het**		29	17.8	Tolerated	0.14	Benign		151156420	0.0006	0.0005	0.00014
Chr10:73464764	A	G	**CDH23**	**Usher syndrome 1D (recessive); Cadherin-like domain**	NM_022124.5	c.2830A>G	p.S944G			**Het**	56	24.3	Damaging	0.01	Probably Damaging	3.82E-04	188098974	0.0006	0.0006	0.0013
Chr10:73567371	G	A	**CDH23**	**Usher syndrome 1D (recessive); Cadherin-like domain**	NM_022124.5	c.8407G>A	p.V2803I		**Het**		29	23.9	Tolerated	0.41	Possibly Damaging	6.56E-05	369697366	0.0004	0.0001	0.00013
Chr10:73574991	C	T	**CDH23**	**Usher syndrome 1D (recessive); No domain**	NM_022124.5	c.10021C>T	p.R3341C			**Het**	180	14.85	Damaging	NA	Possibly Damaging		3700741170	0	0.0001	0.00026
Chr20:368854	G	A	**TRIB3**	**Homolog of Drosophila *Tribbles* , modulator of AKT**	NM_021158.4	c.200G>A	p.R67H		**Het**		29	18.23	Tolerated	0.07	Benign		61746925	0.0032	0.0051	0.0012
Chr20:372180	C	T	**TRIB3**	**Homolog of Drosophila *Tribbles* , modulator of AKT**	NM_021158.4	c.541C>T	p.R181C			**Het**	180	33	Damaging	0.01	Probably Damaging		149447454	0.0004	0.0009	0.0006
Chr22:38109357	C	G	**TRIOBP**	**Deafness 28 (recessive)**	NM_001039141.2	c.395C>G	p.S132C			**Het**	112	6.772	Damaging	NA	Possibly Damaging			0	0	0.000041
Chr22:38130889	C	G	**TRIOBP**	**Deafness 28 (recessive)**	NM_001039141.2	c.4546C>G	p.P1516A	**Het**			27	5.953	Tolerated	NA	Benign			0	0	0
Chr22:38134708	C	G	**TRIOBP**	**Deafness 28 (recessive)**	NM_001039141.2	c.5166C>G	p.D1722E		**Het**		0	14.01	Tolerated	0.19	Benign		183189469	0.0004	0.0003	0.00015
Chr22:38153637	A	C	**TRIOBP**	**Deafness 28 (recessive)**	NM_001039141.2	c.5705A>C	p.K1902T			**Het**	78	12.78	Damaging	0.02	Probably Damaging	2.61E-03	138804394	0.0002	0.0005	0.0013

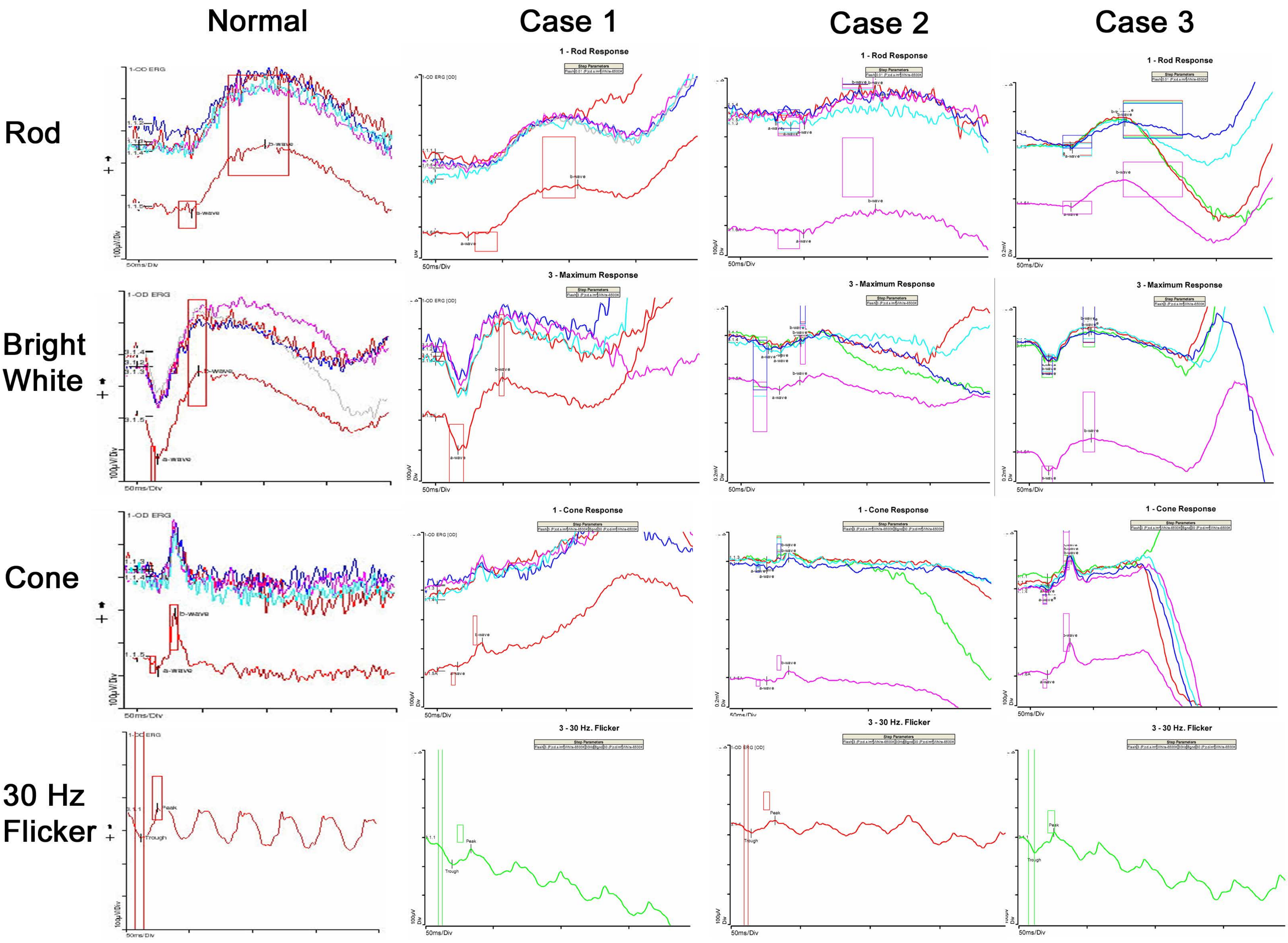
Normal
Case 1
Case 2
Case 3
Rod
Bright White
Cone
30 Hz Flicker
1 - Rod Response
3 - Maximum Response
1 - Cone Response
3 - 30 Hz. Flicker
Step Parameters
a-wave
b-wave
Peak
Trough
1-OD ERG
50ms/Div
100μV/Div
0.2mV Div

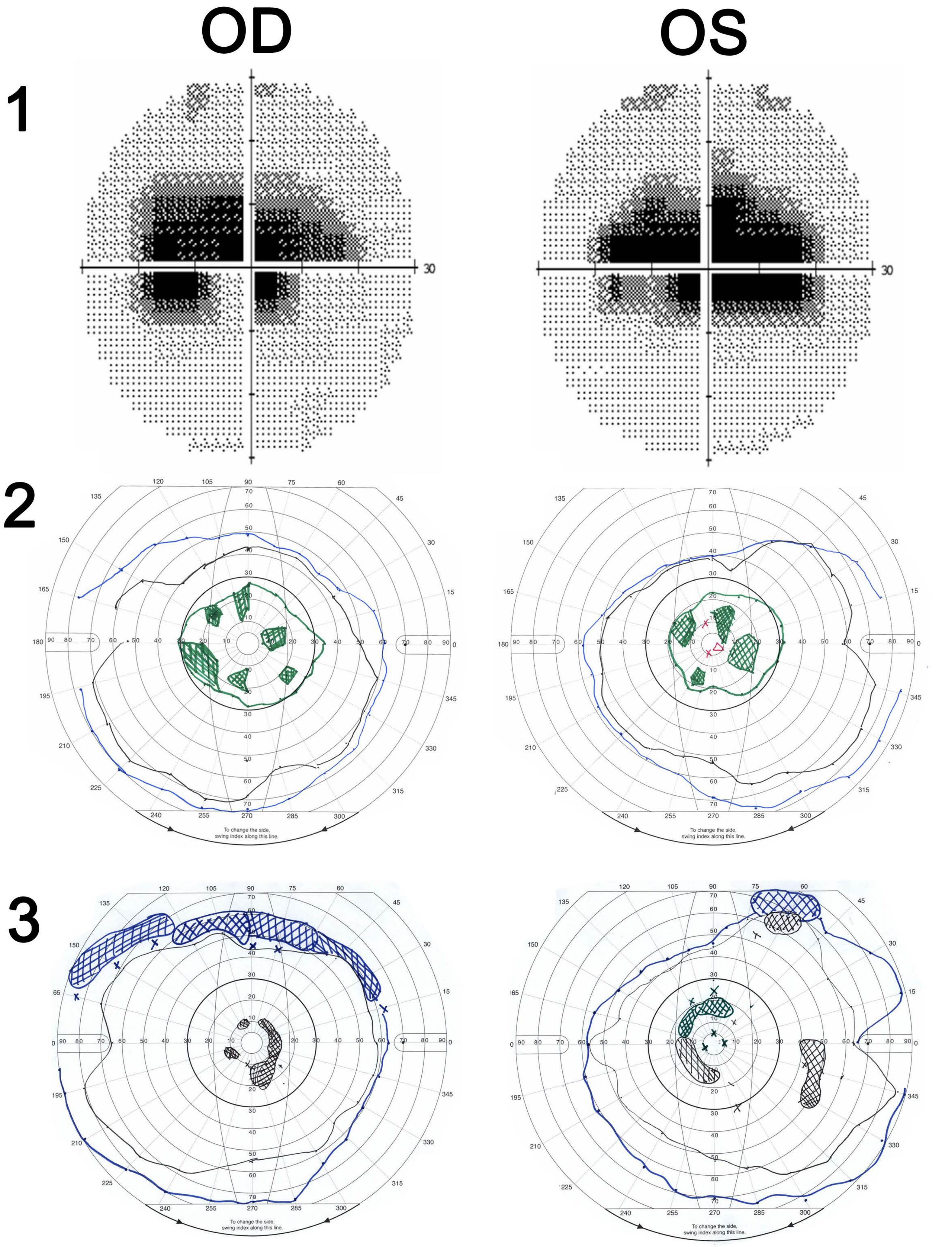

OD
OS
1
2
3
To change the side, swing index along this line.

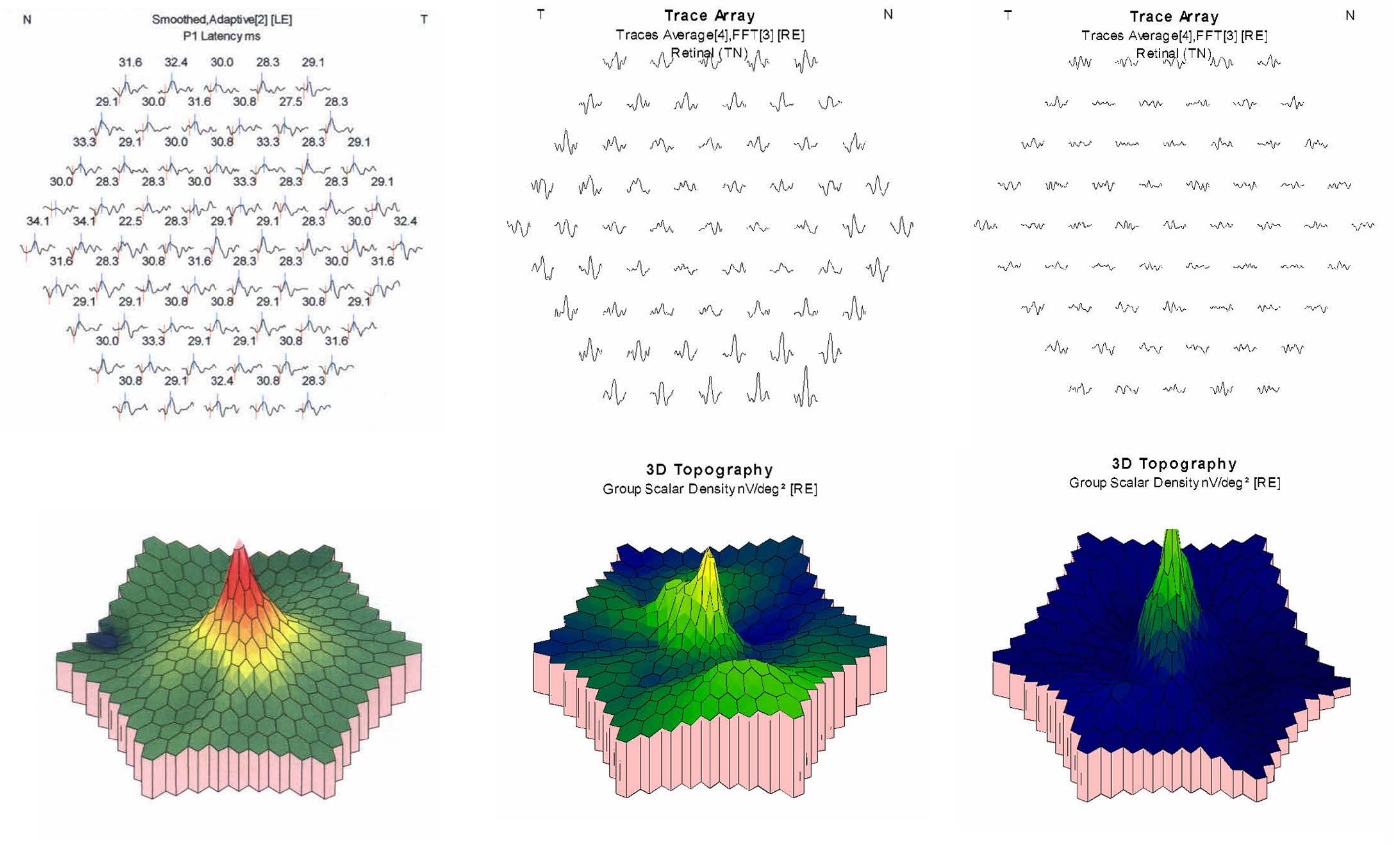

N
Smoothed,Adaptive[2] [LE]
P1 Latency ms
T
31.6 32.4 30.0 28.3 29.1
29.1 30.0 31.6 30.8 27.5 28.3
33.3 29.1 30.0 30.8 33.3 28.3 29.1
30.0 28.3 28.3 30.0 33.3 28.3 28.3 29.1
34.1 34.1 22.5 28.3 29.1 29.1 28.3 30.0 32.4
31.6 28.3 30.8 31.6 28.3 28.3 30.0 31.6
29.1 29.1 30.8 30.8 29.1 30.8 29.1
30.0 33.3 29.1 29.1 30.8 31.6
30.8 29.1 32.4 30.8 28.3
T
Trace Array
Traces Average[4],FFT[3] [RE]
Retinal (TN)
N
T
Trace Array
Traces Average[4],FFT[3] [RE]
Retinal (TN)
N
3D Topography
Group Scalar Density nV/deg² [RE]
3D Topography
Group Scalar Density nV/deg² [RE]
